# Molecular confirmation of the hybrid origin of *Sparganium longifolium* (Typhaceae)

**DOI:** 10.1038/s41598-022-11222-8

**Published:** 2022-05-04

**Authors:** Yinjiao Yu, Fengxia Li, Eugeny A. Belyakov, Weidong Yang, Alexander G. Lapirov, Xinwei Xu

**Affiliations:** 1 National Field Station of Freshwater Ecosystem of Liangzi Lake, College of Life Sciences, Wuhan University, Wuhan, 430072 China; 2 Papanin Institute for Biology of Inland Waters, Russian Academy of Sciences, Borok, Nekouz District, Yaroslavl Region, Russia 152742

**Keywords:** Ecology, Evolution, Plant sciences

## Abstract

*Sparganium longifolium* was reported as a hybrid between *S. emersum* and *S. gramineum* based on its intermediate type or the common characteristics of its parent species. Its hybrid origin needs to be confirmed using molecular technology. We investigated the origin of *S. longifolium* based on 10 populations of *S. emersum*, *S. gramineum* and *S. longifolium* from five lakes in European Russia, using sequences of six nuclear loci and one chloroplast DNA fragment. Haplotype network, principal coordinate analysis and genetic clustering based on data of nuclear loci confirmed that *S. longifolium* is the hybrid between *S. emersum* and *S. gramineum*. We found that the natural hybridization between *S. emersum* and *S. gramineum* is bidirectional but asymmetrical, and the latter mainly acts as maternal species. We also found that all samples of *S. longifolium* were F1 generations, and thus hypothesized that *S. emersum* and *S. gramineum* could likely maintain their species boundary through the post-zygote reproductive isolation mechanism of F1 generation sterility.

## Introduction

*Sparganium* L. (Typhaceae) is an ecologically important group of aquatic plants, comprising about 14 species, widely distributed in the temperate and cold regions of the northern hemisphere^1,2^. Natural hybridization between *Sparganium* species is common. Cook and Nicolls reviewed previous studies and listed different interspecific hybrids in *Sparganium*. All these studies identified hybrids using morphological characteristics. Due to phenotypic plasticity and subtle morphological differences, however, hybrids are often difficult to distinguish from their parents^3^. Therefore, molecular identification is an essential method for the study of natural hybridization in *Sparganium*. Recently, the presence of hybrids, such as *S. angustifolium* Michx. × *S. emersum* Rehmann, *S. fallax* Graebn. × *S. japonicum* Rothert, *S. acaule* Rydb. × *S. fluctuans* B.L.Rob. and *S. glomeratum* Laest. ex Beurl. × *S. gramineum* Georgi was verified based on DNA sequences in two phylogenetic studies^3,4^.

*Sparganium longifolium* Turcz. was reported as a hybrid between *S. emersum* and *S. gramineum* based on their morphological characteristics^1^. This hybrid occurs commonly in regions where both parents grow together and often occupies habitats where *S. gramineum* is absent due to its high adaptability under eutrophic conditions^1^. The hybrid origin of *S. longifolium* was emphasized by^5^ based on their detailed biomorphological investigation. The hybrid was considered fertile^1^ and backcrossing with its parent species was used to explain the phenomenon that some populations of *S. longifolium* were rich in terate forms^5^. So far, only one case study at the molecular level has been conducted for *S. longifolium*. Belyakov et al. sequenced the internal transcribed spacer and found similar and identical ribotypes in *S. emersum*, *S. longifolium*, *S. gramineum* and *S. hyperboreum*, which did not provide directly molecular evidence to clarify the origin of *S. longifolium*^6^. Further molecular studies are still necessary to confirm the hybrid origin of *S. longifolium*. In addition, the direction of hybridization is unknown. If a bidirectional hybridization exists, two distinct life forms of *S. longifolium*, emergent and floating-leaved, which are similar to *S. emersum* and *S. gramineum* respectively, are likely correlated to the direction of hybridization. All these hypotheses need to be verified using molecular technology.

In this study, we collected samples of *S. emersum*, *S. gramineum* and *S. longifolium* from five lakes in European Russia, using sequences of six nuclear loci and one chloroplast DNA fragment to (1) test whether *S. longifolium* is the hybrid between *S. emersum* and *S. gramineum*, and (2) detect the direction of hybridization. The study will deepen our understanding of interspecific hybridization in *Sparganium*.

## Results

### Sequence variations

**Figure 1:**
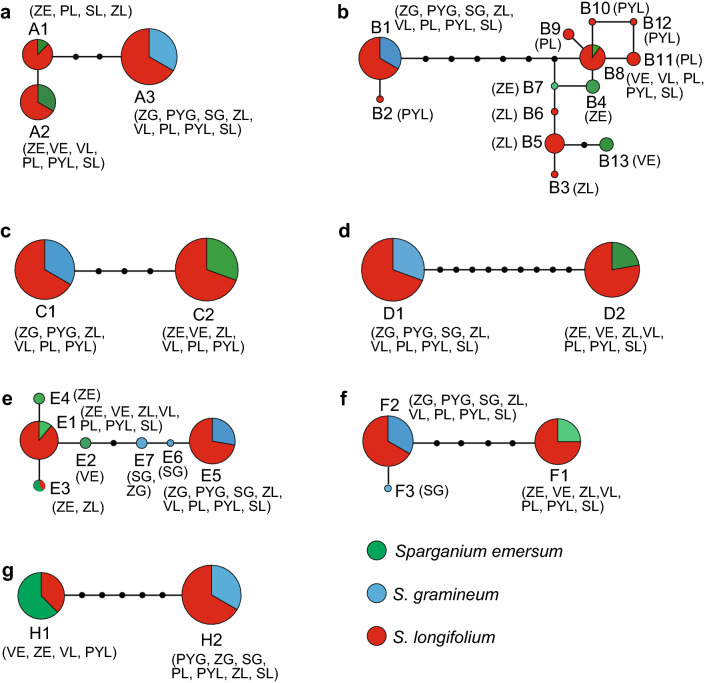
Haplotype networks of six nuclear loci Tran05 (**a**), Tran57 (**b**), Tran59 (**c**), Tran66 (**d**), Tran83 (**e**), and Tran93 (**f**), and cpDNA fragment *trn*H-*psb*A (**g**). Node size is proportional to the number of each haplotype. Small black circles represent unsampled or hypothetical haplotypes. Population codes indicated beside haplotypes are the same as Table 1.

Sequences of the six nuclear loci were obtained from 85, 83, 62, 85, 84 and 86 individuals, respectively. Their aligned lengths were 579, 492, 501, 465, 326 and 413 bp with 4, 14, 4, 10, 7 and 6 variable sites, respectively (Supplementary Table S1). The numbers of haplotypes at the six nuclear loci were 3, 13, 2, 2, 7 and 3, respectively. The haplotype networks of the six nuclear loci showed the same pattern: haplotypes of *S. emersum* and *S. gramineum* separated well and formed two clades, while all individuals of *S. longifolium* were heterozygous and consisted of two alleles from different haplotype clades (Fig. 1). Of the six nuclear loci, only at the Tran57 locus *S. longifolium* had private haplotypes (Fig. 1).

Sequences of *trn*H-*psb*A were obtained from 82 individuals, including 15 of *S. emersum*, 20 of *S. gramineum* and 47 of *S. longifolium*. The aligned length was 670 bp with four substitutions and two 4-bp indels. Sequences of all samples collapsed into two haplotypes H1 and H2 corresponding to *S. emersum* and *S. gramineum*, respectively (Fig. 1g). In *S. longifolium*, nine individuals from VL and PYL populations shared H1 with *S. emersum* and 38 individuals from PL, PYL, ZL and SL populations shared H2 with *S. gramineum* (Fig. 1g).

### Genetic grouping

**Figure 2:**
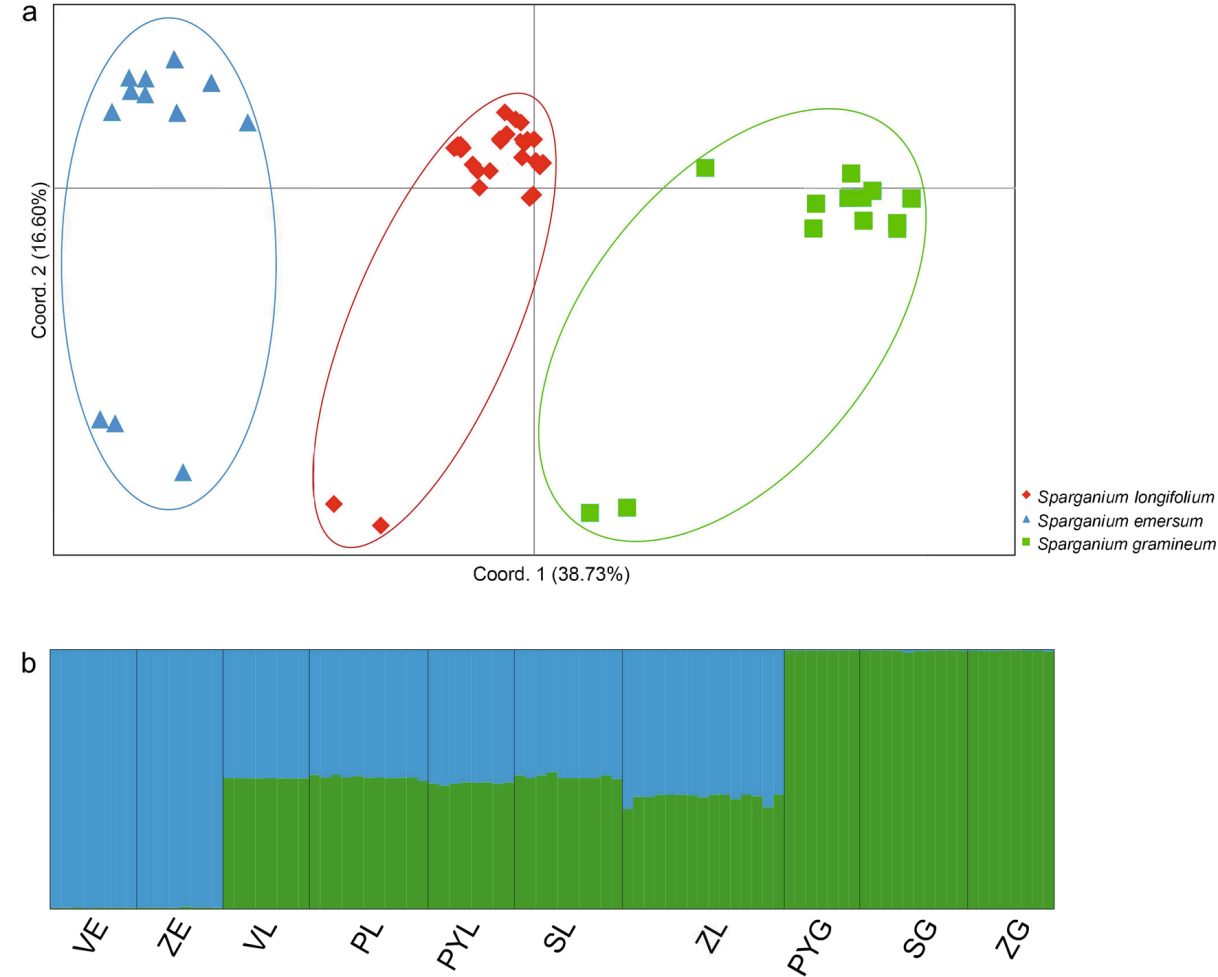
Genetic clustering for 10 populations of *Sparganium emersum*, *S*. *longifolium* and *S. gramineum* using principal coordinate analysis (**a**) and STRUCTURE (**b**) based on six nuclear loci. In (**b**), blue and green represent the geneic clusters of *S. emersum* and *S. gramineum*, respectively, and a single vertical bar displays the membership coefficients of each individual. Population codes follow Table 1.

The PCoA analysis revealed that all samples were divided into three groups on principal coordinate 1, which explained 38.73% of the total variation. The three groups corresponded to *S. emersum*, *S. gramineum* and *S. longifolium*, respectively, and the hybrid group was located between two parent groups (Fig. 2a). STRUCTURE analysis suggested K = 2 as the optimal number of clusters based on the value of ΔK (Supplementary Fig. S1) and inferred two genetic clusters that consisted of *S. emersum* and *S. gramineum* respectively and genetic admixture for all samples of *S. longifolium* with intermediate admixture coefficient (0.3863–0.5279, Fig. 2b).

## Discussion

The haplotype networks, PCoA analysis and STRUCTURE analysis based on the six nuclear loci confirm that *S. longifolium* is a hybrid between *S. emersum* and *S. gramineum*, providing molecular support for previous morphological analyses^5^. Furthermore, all individuals with intermediate admixture coefficient (Fig. 2b) and private haplotypes only present in one out of six nuclear loci (Fig. 1) suggest that *S. longifolium* is most likely a F1 hybrid. We thus hypothesized that *S. emersum* and *S. gramineum* could likely maintain their species boundary through the post-zygote reproductive isolation mechanism of F1 generation sterility. This hypothesis is possible based on the observations from hybrids in European Russia. The pollen viability was checked in *S. longifolium* samples from Vysokovskoe Lake and Sabro Lake, and the vast majority of checked pollens were sterile^5^. In addition, flowering plants of *S. longifolium* often do not form seeds, or the seeds are puny and significantly inferior to normal seeds in size^5^. However, the hypothesis is only based on our limited sampling, which is contrary to the conclusion inferred from morphological characteristics that it is fertile and may backcross with parental species^1^. Further studies with extensive sampling are necessary to test our hypothesis.

The chloroplast DNA fragment *trn*H-*psb*A was used to infer the direction of hybridization between *S. emersum* and *S. gramineum* because chloroplast DNA is maternal inheritance in *Sparganium*^3,4^. The hybrid *S. longifolium* shared haplotypes with *S. emersum* and *S. gramineum* simultaneously (Fig. 1). This finding clearly indicates that bidirectional hybridization exists between *S. emersum* and *S. gramineum*. At the same time, the different frequency of these two haplotypes in the hybrid (H1, 19.1% vs. H2, 80.9%) means that the direction of hybridization is asymmetric. A variety of factors can lead to asymmetry in natural hybridization, such as flowering time, preference of pollinators, quality and quantity of pollen, cross incompatibility and the abundance of parent species^7,8^. Rare species usually act as maternal species relative to abundant species^9,10^. *S. gramineum* is confined to oligotrophic lakes and its abundance is obviously lower than that of *S. emersum*^1,11^. The relatively scarcity combined with the ecology of *S. gramineum* make it more often act as maternal species when hybridizing with *S. emersum*.

As described by^5^, the morphological diversification of *S. longifolium* was also observed in this study. For example, individuals of *S. longifolium* with emergent and floating-leaved life forms occur concurrently in Zaozer’ye Lake (Supplementary Fig. S2). However, all individuals had the same haplotype H2 as *S. gramineum* (Fig. 1), suggesting that the direction of hybridization do not determine life form of *S. longifolium*. In addition, all individuals of *S. longifolium* sampled here are likely F1 hybrid. Their variable phenotypes could not be associated with traits segregation due to F2 generation or backcross. Detailed ecological investigation combining with research at the genomic level are essential to find out the potential factors leading to morphological diversification of *S. longifolium*.

Here, using sequences of six nuclear loci and one chloroplast DNA fragment, we confirmed that *S. longifolium* is the hybrid between *S. emersum* and *S. gramineum*. The natural hybridization between *S. emersum* and *S. gramineum* is bidirectional but the latter mainly acts as maternal species. We also found that all samples of *S. longifolium* were F1 generations, indicating that *S. emersum* and *S. gramineum* could maintain their species boundary through the post-zygote reproductive isolation mechanism of F1 generation sterility.

## Methods

### Sample collection and DNA extraction

A total of 93 individuals from 10 populations of *S. emersum*, *S. gramineum* and *S. longifolium* were collected from five lakes in European Russia (Table 1). Individuals of each population were collected randomly at intervals of at least 10 m. The collection of plant materials was approved by Papanin Institute for Biology of Inland Water (IBIW), Russian Academy of Sciences. Voucher specimens were kept in the herbarium of IBIW and identified by Dr. Eugeny A. Belyakov (IBIW). Fresh leaves were sampled and dried in silica gel for subsequent DNA extraction. Total genomic DNA was extracted using the DNA Secure Plant Kit (Tiangen Biotech, Beijing, China) following the manufacturer’s protocol.

**Table 1:** Sampling sites and number of samples for *Sparganium emersum*, *S. longifolium* and *S. gramineum*.

Code	Species	n	Collection no.	Location	Latitude/longitude	Collection date
VE	*S. emersum*	8	Be1	Vysokovskoe Lake, Ivanovo	57.1726/40.9405	2018/8/31
VL	*S. longifolium*	8	Be2	Vysokovskoe Lake, Ivanovo	57.1726/40.9405	2018/8/31
PL	*S. longifolium*	11	Be4	Polevo Lake, Ivanovo	56.5509/41.5945	2018/9/1
PYG	*S. gramineum*	7	Be5	Pyrskoe Lake, Nizhny Novgorod	56.3970/43.3074	2018/9/7
PYL	*S. longifolium*	8	Be6	Pyrskoe Lake, Nizhny Novgorod	56.3970/43.3074	2018/9/7
ZG	*S. gramineum*	8	Ru114	Zaozer’ye Lake, Yaroslavl	56.8248/39.3571	2018/8/21
ZL	*S. longifolium*	15	Ru115	Zaozer’ye Lake, Yaroslavl	56.8248/39.3571	2018/8/21
ZE	*S. emersum*	8	Ru116	Zaozer’ye Lake, Yaroslavl	56.8248/39.3571	2018/8/21
SG	*S. gramineum*	10	Ru096	Sabro Lake, Tver	57.1634/32.9105	2018/8/17
SL	*S. longifolium*	10	Ru097	Sabro Lake, Tver	57.1634/32.9105	2018/8/17

### Amplification, sequencing and cloning

We sequenced one chloroplast DNA fragment *trn*H-*psb*A^12^ and six nuclear loci developed from transcriptome sequences of *S. fallax* (Supplementary Table S1). PCR reactions, sequencing and cloning were performed following^13^. All individuals of *S. longifolium* were heterozygous at the six nuclear loci with multi-point mutations or insertions/deletions, and their alleles were obtained by cloning. Sequence data were aligned using MAFFT v7.3.1^14^.

### Data analyses

Haplotypes of each locus were identified using DNASP v5.0^15^. The obtained haplotypes were deposited in GenBank (see Supplementary Table S1 for accession numbers). A median-joining network^16^ to interpret relationships among haplotypes of each locus was generated using NETWORK v4.0 (http://www.fluxus-engineering.com). The following analyses were performed based on the dataset of six nuclear loci. The genetic lineage proportion of each individual was identified using a Bayesian clustering method implemented in STRUCTURE v2.4^17^. We performed 10 replicate runs with a burn-in period of 20,000 iterations and 100,000 Markov Chain Monte Carlo (MCMC) iterations under the admixture model at the number of clusters from one to eight. Principal coordinate analysis (PCoA) implemented in GenALEx v6.5^18^, was also used to examine the genetic clusters of all individuals.

All methods were performed in accordance with the relevant guidelines and regulations.

## Supplementary Information

Supplementary Information.

## Data Availability

The data that support the findings of this study are available in the GenBank with accession numbers ON015932-ON015962, [https://www.ncbi.nlm.nih.gov/].
