# Supplementary material for: Molecular confirmation of the hybrid origin of Sparganium longifolium (Typhaceae)
Source: Sci Rep. 2022 May 4;12:7279. doi: 10.1038/s41598-022-11222-8 (PMC9068738; doi:10.1038/s41598-022-11222-8)
Supplement: Supplementary file 1 — Supplementary Information. [file 41598_2022_11222_MOESM1_ESM.docx]

**Supplementary Information**

**Molecular confirmation of the hybrid origin of *Sparganium longifolium* (Typhaceae)**

Yinjiao Yu, Fengxia Li, Eugeny A. Belyakov, Weidong Yang, Alexander G. Lapirov, Xinwei Xu

Authors for correspondence: Xinwei Xu, National Field Station of Freshwater Ecosystem of Liangzi Lake, College of Life Sciences, Wuhan University, China. E-mail: [xuxw@whu.edu.cn](mailto:xuxw@whu.edu.cn)

Alexander G. Lapirov, Papanin Institute for Biology of Inland Waters Russian Academy of Sciences, Russia. E-mail: [a_lapir@ibiw.ru](mailto:a_lapir@ibiw.ru)

**Table S1** Primers of six nuclear loci and *trn*H-*psb*A and accession numbers of haplotype sequences deposited in GenBank.

|  | Primers (5’-3’) | Predicted protein | Aligned length (bp) | Variable sites (indel) | Accession number of haplotypes |
| --- | --- | --- | --- | --- | --- |
| Tran05 | F: ATGGTTTGGAGCATACTGT  R: GCAAGACCCTTACGATTT | NEP1-interacting protein | 579 | 4 (1) | A1: ON015932; A2: ON015933; A3: ON015934 |
| Tran57 | F: GACGCCAAACACCTTCGAT  R: CTCGGCTCGGTTACACTC | peroxidase | 492 | 14 (5) | B1: ON015935; B2: ON015936; B3: ON015937;  B4: ON015938; B5: ON015939; B6: ON015940;  B7: ON015941; B8: ON015942; B9: ON015943;  B10: ON015944; B11: ON015945; B12: ON015946;  B13: ON015947 |
| Tran59 | F: TTACTCCATACTGCCGCTA  R: TATGCCCATGAAGATACACA | ent-kaurene oxidase | 501 | 4 (1) | C1: ON015948; C2: ON015949 |
| Tran66 | F: TACAAACAACCTCCCACT  R: AACAGCACCATCTACTCC | ribokinase | 465 | 7 | D1: ON015950; D2: ON015951 |
| Tran83 | F: AGGCATCTTTGGGTGGTA  R: GACTTGCGTGCTGTTGTG | uncharacterized  protein | 326 | 7 | E1: ON015952; E2: ON015953; E3: ON015954;  E4: ON015955; E5: ON015956; E6: ON015957;  E7: ON015958 |
| Tran93 | F: AGACTTGAACTAAGCACCAC  R: AGAAGACCCGACACTACC | 60S ribosomal  protein | 413 | 6 | F1: ON015959; F2: ON015960; F3: ON015961 |
| *trn*H- *psb*A | F: CGCGCATGGTGGATTCACAATCC  R: GTTATGCATGAACGTAATGCTC |  | 670 | 6 (2) | H1: ON015962; H2: ON015963 |


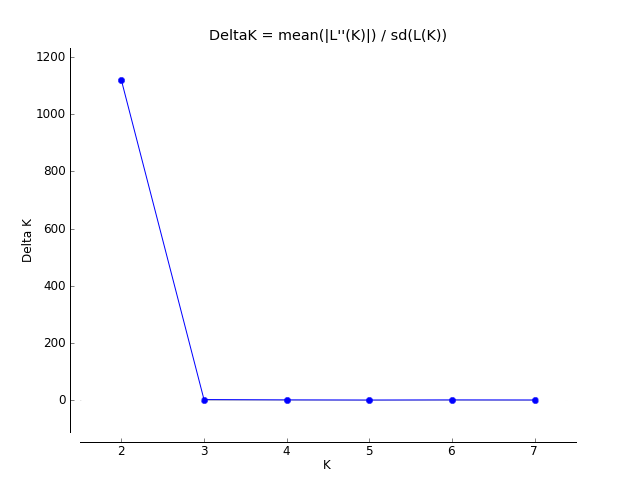


**Figure S1** Modelling of the number of genetic clusters in 10 populations of *Sparganium emersum*, *S*. *longifolium* and *S. gramineum* using STRUCTURE. Delta K (ΔK) calculated based on Evanno et al. (2005), charted against the number of modeled genepools (K).


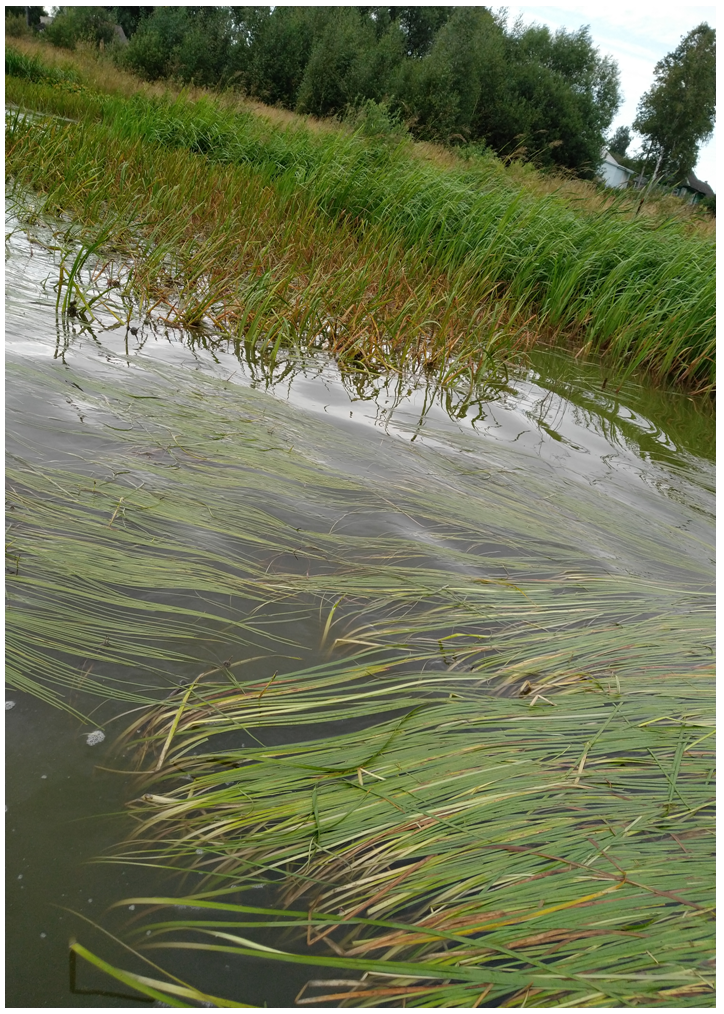


**D**

**C**

**B**

**C**


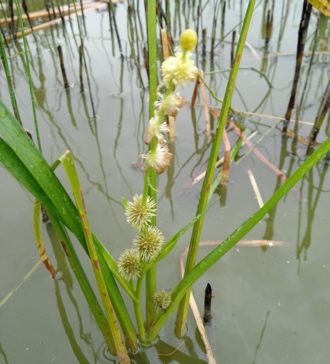

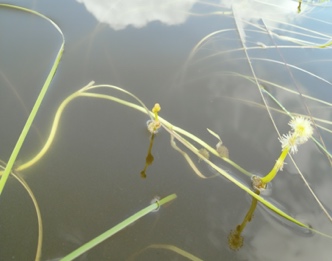

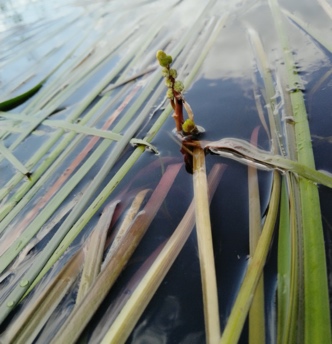

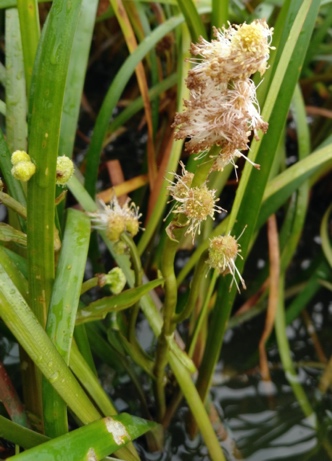


**A**

**B**

**E**

**A**

**D**

**Figure S2** Sympatric distribution of *Sparganium* *longifolium* and its two parent species in Zaozer’ye Lake, Yaroslavl, Russia. A: *S. emersum*; B: *S*. *gramineum*; C: emergent form of *S*. *longifolium*; D: floating-leaved form of *S*. *longifolium*; E: habitats.
